# Disseminated Gastric MALT Lymphoma with Monoclonal Gammopathy, t(11;18)(q21;q21), and Subsequent Development of T-Large Granular Lymphocytic Leukemia: A Case Report and Review of the Literature

**DOI:** 10.1155/2015/953297

**Published:** 2015-05-06

**Authors:** Riad Akoum, Wassim Serhal, Hussein Farhat

**Affiliations:** ^1^Division of Medical Oncology, Lebanese American University Medical Center-Rizk Hospital, Beirut 11-3288, Lebanon; ^2^Department of Laboratory Medicine and Pathology, Lebanese American University Medical Center-Rizk Hospital, Beirut 11-3288, Lebanon

## Abstract

*Background*. Extranodal marginal zone lymphoma of mucosa-associated lymphoid tissue (MALT) is a well-characterized entity that may share clinical and morphological findings with other low-grade non-Hodgkin's lymphomas. Dissemination of MALT-type lymphoma to bone marrow and peripheral blood simultaneously with the presence of T-large granular cell leukemia (T-LGL) has rarely been reported. *Case Presentation*. This is the case of a 42-year-old male who presented with a gastric MALT-type lymphoma, disseminated to the bone marrow and the peripheral blood with high serum IgM levels and t(11;18)(q21;q21). The morphological, immunophenotypical and, immunohistochemical studies of the successive bone marrow and peripheral blood samples had revealed the coexistence of two distinct lymphoma cell populations: a B-cell, marginal zone type population expressing CD19, CD20, CD22, CD79b, IgM, and kappa light chain, and a T-large granular cell population, developed after treatment with rituximab expressing CD3, CD8, CD5, CD7, and CD45. *Conclusion*. Based on the analysis of this unusual case we performed an extensive review of the literature to elucidate the relationship between T-LGL and B-cell lymphomas and to emphasize the importance of paraprotein analysis at diagnosis of gastric MALT lymphoma.

## 1. Introduction

Mucosa-associated lymphoid tissue (MALT) lymphomas are extranodal B-cell marginal zone lymphomas that generally follow an indolent course. Fifty percent of all MALT lymphomas arise from the stomach and are commonly associated with* Helicobacter pylori* infection. Nongastric MALT lymphomas occur in the lung, salivary gland, skin, and other organs often associated with autoimmune disease. Bone marrow involvement has been reported in 23.5% to 37% of cases at presentation [1–3]. Leukemic dissemination has only sporadically been reported [4, 5].

Disseminated MALT lymphoma may mimic Waldenstrom macroglobulinemia by causing Waldenstrom syndrome. Monoclonal gammopathy (MG) was detected in 17.2% of cases with B-cell NHL [6] and 36% of cases with MALT-type lymphoma [7]. IgG was more frequent in cases with aggressive NHL, while IgM was more common in cases with low-grade NHL. It was usually associated with advanced disease, typically showing bone marrow and peripheral blood involvement (Table 1) [8–10]. However, Wöhrer et al. found that MG although a common phenomenon in MALT lymphoma was not correlated with clinical stage, genetic findings,* H. pylori* status, or response to treatment [7].

CD5 expression is rare in MALT lymphoma and is often associated with nongastric disease and an increased tendency to present with disseminated disease [11].

The detection of t(11;18)(q21;q21) is useful in disseminated cases. This translocation was predominantly found in gastric MALT lymphoma [12], associated with the resistance to* H. pylori* eradication therapy [13, 14] and associated with the development of* H. pylori*-independent gastric MALT lymphoma [15].

Concomitant or sequential occurrence of MALT lymphoma and other primary B-cell neoplasms has been reported [53]. Coexistence of B-cell and T-cell lymphomatous populations in the same patient has rarely been reported [54, 55]. However, association of T-cell leukemia and MALT lymphoma had not yet been described.

We describe in the present report a case of disseminated gastric MALT lymphoma, with t(11;18)(q21;q21), MG, resistance to* H. pylori* eradication, chemotherapy and immunotherapy, and subsequent appearance of a predominant T-cell large granular leukemia.

## 2. Case Report

A 42-year-old male/bricklayer was admitted in 2006 for high grade fever and dyspnea. He had a 2-month history of epigastric pain, peptic discomfort, and dizziness. Physical examination showed left pleural effusion, ascites, dehydration, and cachectic appearance. Laboratory tests revealed a hemoglobin level of 10.5 g/dL, a white blood cell count of 21.3 Giga/L with 80% neutrophils and 12% atypical lymphocytes, a total serum protein level of 101 g/L with hypoalbuminemia at 30 g/L (N: 40–47 g/L), an IgM level of 52 g/L (0.5–2.4 g/L), a kappa light chains level of 9.07 g/L (N: 2–4.4 g/L), a kappa/lambda ratio of 7.96 (N: 1.35–2.65), and a C reactive protein (CRP) of 365 mg/L (N: <5 mg/L); the Bence-Jones protein in urine was negative; the renal and liver function tests, LDH, and *β*2-microglobulin titers were normal. The serologic tests for EBV, CMV, and chronic viral hepatitis including HCV were unremarkable. The total body CT scan showed a left pleural effusion with lower lobe atelectasis, circumferential thickening of the gastric wall predominantly affecting the greater curvature. There was no hepatomegaly, splenomegaly, or brain lesion. A total skeletal survey showed no bone lesion. The bronchial endoscopy showed no tumor. Gastric endoscopy confirmed the presence of a huge tumor with surface ulceration at the greater curvature. Histological examination of biopsies revealed a typical lymphoepithelial lesion compatible with low-grade MALT-type lymphoma and positive* Helicobacter pylori* chronic gastritis. The immunostaining showed positive CD20, CD5, CD38, and *κ*-light chain stains but negative CD10, cyclin D1, and *λ*-light chain stains. The bone marrow aspirate and biopsy showed colonization with plasmacytoid cells and dense infiltrations by small lymphocytes extending to the paratrabecular zone. The karyotype study of the bone marrow aspirate revealed a typical, specific translocation of gastric MALT lymphoma, t(11;18)(q21;q21) in 30 out of 38 metaphases (Figure 3).

The cytological examination of the pleural and peritoneal fluids was negative. The histological examination of the pleural biopsy showed a nonspecific subacute suppurative pleuritis with no evidence of malignancy.

**Figure 1:**
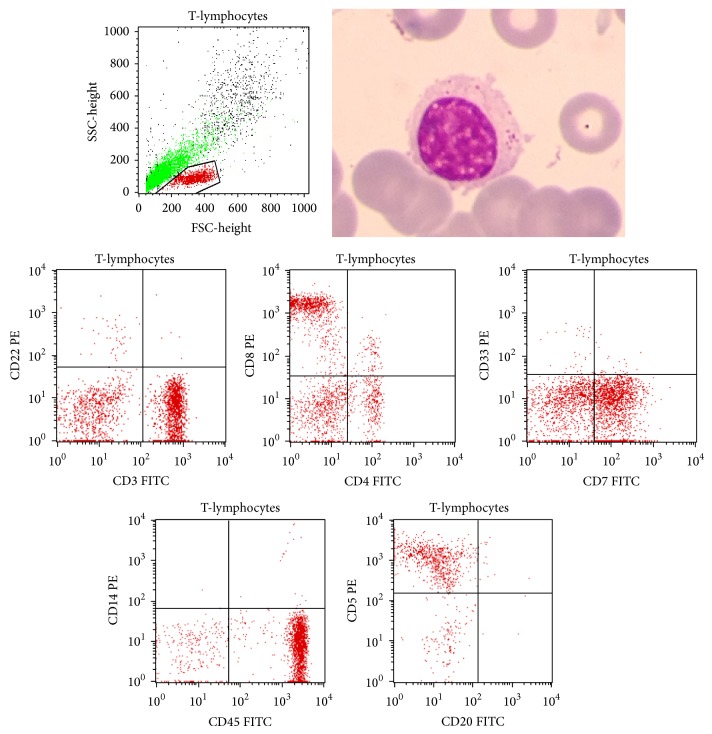
A T-lymphocyte showing an abundant granular cytoplasm and selected gated dual parameter dot plots of T-cells displaying CD3, CD8, CD7, CD45, and CD5.

After adequate hydration and antibiotic therapy, the respiratory function and the white blood cell count returned to normal but with an atypical plasmacytoid lymphocytosis reaching 35%. The patient received 6 courses of cyclophosphamide, fludarabine, and rituximab and then 3 courses of cisplatin-based chemotherapy. The gastric lesion and the monoclonal paraproteinemia remained, however, unchanged. The cytological examination of the bone marrow aspirate revealed the presence of a predominant mature granular lymphocytosis associated with an atypical plasmacytoid lymphocytosis. The flow cytometry analysis of this aspirate identified two cell populations, one population of T-cells expressing mainly the CD8+/CD3+/CD5+/CD7+/CD45+ immunophenotype representing 40% of the examined cells (Figure 1) and a second population representing 20% of these cells and consisting of monoclonal B-cells expressing kappa light chain, IgM, CD19, CD79b, CD20, and CD22.

The patient was ultimately put on an expectant management option “watchful waiting.” The serum electrophoresis peak remained the same (Figure 4); the gastric lesion remained unchanged during four years. The patient died from an evolving pulmonary infection in 2013.

Molecular study of T-cell receptor genes was attempted in postmortem using the paraffin-embedded bone marrow specimen that has failed to assess clonality because of the degraded DNA.

## 3. Discussion

The leukemic presentation, the refractoriness to chemoimmunotherapy, the IgM kappa production, and the presence of t(11;18)(q21;q21) characterize the clinical picture of our patient with gastric MALT lymphoma. Therefore, the marginal zone B-cell population invading the bone marrow and the peripheral blood has decreased, after treatment with rituximab, and a second malignant T-cell population emerged and became predominant.

**Figure 2:**
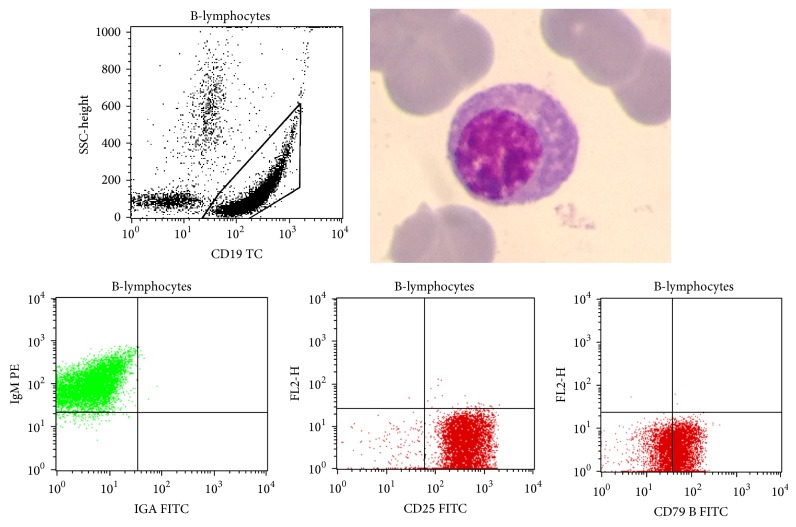
A B-lymphocyte showing a plasmacytic differentiation and selected gated dual parameter dot plots displaying CD19, IgM, CD25, and CD79a.

**Figure 3:**
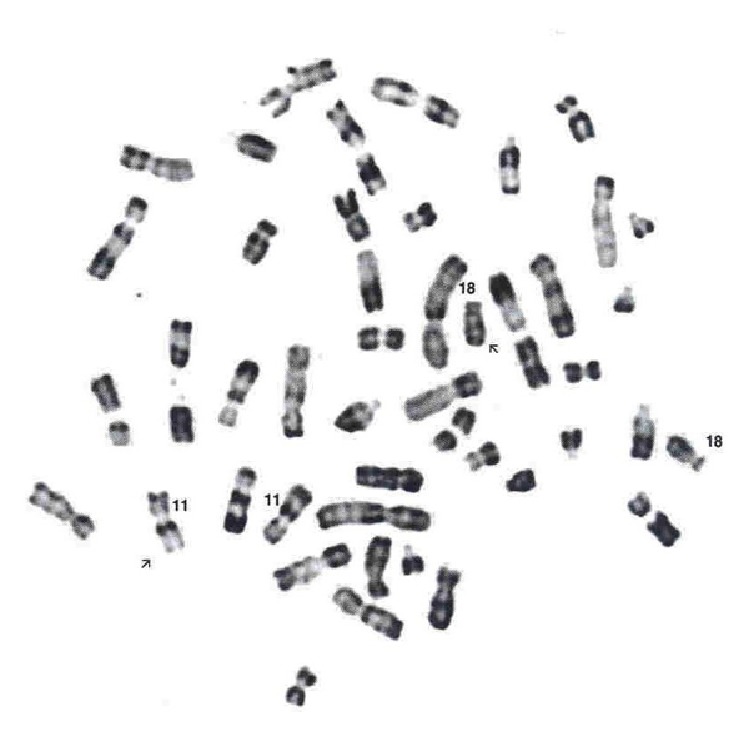
The karyotype of the malignant bone marrow cells at presentation that shows the t(11;18)(q21;q21).

**Figure 4:**
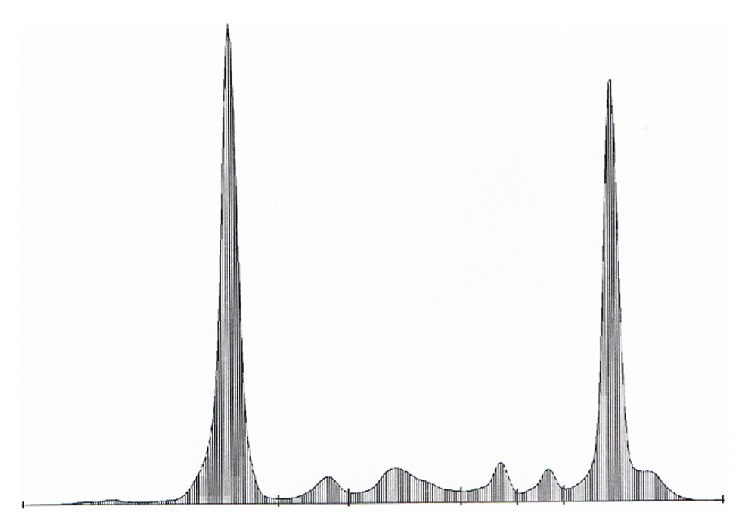
The serum protein electrophoresis graph that remained unchanged during the course of the disease.

The tumor cells at the initial presentation had plasmacytic differentiation (Figure 2). The plasmacytic morphology may be found in 30% of extragastric MALT-type lymphoma [7] and only 10% of gastric MALT lymphoma [56, 57]. It is a rare finding in lymphomas with t(11;18)(q21;q21) which are mostly associated with monocytoid morphology [56].

Leukemic dissemination has only sporadically been described in MALT lymphoma [4, 5]. It has been significantly related to bone marrow infiltration [58]. However, the presence of a serum monoclonal component has not been associated with the disseminated disease [6].

The translocation t(11;18)(q21;q21) is the most structural chromosomal abnormality found in MALT-type lymphoma occurring in about one-third of the cases, involving different sites mainly gastric ones, at any stage [58]. It has been shown that this translocation is a marker of resistance to* H. pylori* eradication and may indicate that it confers an independent growth advantage [59]. The trisomy of chromosome 3 represents the most frequent numerical abnormality in MALT lymphoma; however, it is not specific for this lymphoma subtype and has no prognostic significance although it has been associated with a plasmacytoid appearance of the leukemic lymphocytes and IgM hypergammaglobulinemia [60]. Although t(14;18)(q32;q21)/IGH-BCL2 is the genetic hallmark of follicular lymphoma, this reciprocal translocation, closely related to t(11;18), has been described in extranodal marginal zone lymphoma, mostly nongastric MALT lymphoma [58]. BCL10 nuclear expression is also closely related to the presence of the t(11;18) and found in disseminated gastric MALT lymphoma [13, 15].

Gastric MALT lymphoma with t(11;18) and extragastric MALT lymphoma with trisomy 18 are groups with the higher risk of dissemination [61].

CD5 expression is typically absent in MALT-type lymphoma; however, it is sometimes aberrantly coexpressed in nongastric, even localized disease [62] and associated with increased tendency to relapse, refractoriness to therapy, and dissemination to bone marrow [11, 63]. It has also been associated with monoclonal paraprotein production in some cases (Table 2).

Positive expression of BCL2 has been associated with unfavorable survival in extranodal diffuse large B-cell lymphomas (DLBCL) and MALT lymphomas [64].

The frequency of* H. pylori* infection is higher in MALT lymphoma restricted to the stomach. Although the eradication of* H. pylori* may result in clinical and histological remission in 90% of patients, molecular evidence of persistent gastric MALT lymphoma may be found in 40% of these cases [65].

The curative potential of chemotherapy and immunotherapy is questionable [66]. Plasmacytic differentiation and monoclonal gammopathy do not influence the rate of disease progression. Rituximab has only moderate activity in terms of inducing objective responses in disseminated MALT lymphoma. However, long-term disease stabilization along with a symptomatic benefit has been seen in some patients [61]. Moreover rituximab could select latent clonal CD20− populations in some patients.

The morphology and the immunophenotype CD3+/CD5+/CD7+/CD45+/CD10− of the second malignant population in the bone marrow of the presented patient are consistent with the sequential occurrence of a T-large granular cell leukemia. The association of clonal T-LGL proliferations with clonal B-cell lymphoproliferative disorders, although rare, is now well recognized [54, 67]. T-LGL is a chronic and often indolent T-cell proliferation. The transformation of an indolent lymphoma to a more aggressive one of the same immunological origin is a well-recognized event. In a population-based series of unselected patients with multiple histology lymphomas, Tucci et al. [53] reported that the most frequent transformation from marginal zone lymphoma was to DLBCL. Reciprocally a sequential appearance of marginal zone lymphoma after treatment for DLBCL has also been observed which may have been unrecognized in the first diagnostic biopsy. Coexistence of B-cell and T-cell lymphoma populations in the bone marrow and peripheral blood of the same patient has rarely been reported. Synchronous clonal T-LGL has been reported in patients with splenic marginal zone lymphoma [54, 55].

Reported cases of gastric and nongastric MALT-type lymphoma with monoclonal gammopathy are summarized in Tables 1 and 2. Thymic MALT lymphoma seems to be clinicopathologically a distinctive form with prevalence in Asians, strong association with autoimmune disease, marked female predominance, frequent presence of epithelium-lined cysts, almost invariable presence of a neoplastic plasma cell component, expression of IgA phenotype, and absence of API2-MALT1 gene fusion [68, 69]. Cases of chronic autoimmune thyroiditis (Hashimoto's thyroiditis) have been reported in patients with MALT lymphoma. Most of these patients had tumors with plasmacytic differentiation and two of them presented with monoclonal gammopathy [70].

## 4. Conclusion

Leukemic dissemination and monoclonal macroglobulinemia have only sporadically been described in MALT-type lymphoma. Furthermore, subsequent development of T-cell LGL with simultaneous presence of two different lymphoma populations in the peripheral blood and the bone marrow remains an unusual event. Thus, pending additional data, we recommend including the paraprotein analysis and the flow cytometric studies in the pretherapeutic workup of patients with MALT lymphoma.

## Figures and Tables

**Table 1:** Reported cases of gastric MALT lymphoma with monoclonal gammopathy.

Author/year	Age	Sex	*H pylori *	Dissemination	Serum Ig	Genetic findings	Bcl-2	Reference
Levine et al./1989	54	F	−	None	IgM, IgD, *λ*	T(11;18)		[16]
Allez et al./1999	31	M	−	BM	IgM*κ*	Tri 3		[17]
Griesser et al./1990	46	F	NA	BM	IgM*κ*	NA	+	[5]
Leroux et al./1993	58	M	−	GN	IgA*λ*	T(11;18)		[18]
Hirase et al./2000	77	M	−	BM, PB	IgM*κ*	T(11;18)	−	[8]
	57	F	−	BM, PB	IgM*λ*	T(11;18)	−	
Iwase et al./2000	80	M	NA	BM	IgM*κ*	NK		[19]
Okada et al./2001	77	F	−	BM	IgM*λ*	NK		[20]
Valdez et al./2001	79	M	+	BM	IgM*λ*	NA		[9]
Kunisaki et al./2003	66	M	NA	BM, PB, PE	IgM*κ*	T(11;18)		[21]
Wöhrer et al./2004	90	M	+	None	IgG*κ*	NK		[22]
	55	M	+	BM, PB	IgA*κ*	T(11;18)		
Ye et al./2004	78	F	−	BM, PB	IgM*λ*	Bcl-10		[23]
Gimeno et al./2006	69	F	+	None	IgM*κ*	NK		[24]
Lantuejoul et al./2007	50	F	+	BM, lung	Ig *λ*	NA		[25]
Salle et al./2007	59	M	NA	None	IgM*κ*	NA		[26]
Ohno and Isoda/2008	55	M	+	BM, PB	IgA*κ*	T(11;18)		[27]
Almehmi and Fields/2009	66	F	+	None	IgM*κ*	NK	+	[28]
Reitter et al./2010	35	F	+	BM, PB	IgM*λ*	Tri 3q,18q	+	[4]
Hirota-Kawadobora et al./2012	70	M	+	BM	IgM	NK		[29]
Wu et al./2014	51	M	NA	None	IgA*λ*	NA		[30]

NA: not available; NK: normal karyotype; BM: bone marrow; PB: peripheral blood; PE: pleural effusion.

**Table 2:** Reported cases of nongastric MALT lymphoma with monoclonal gammopathy.

Author/year	Age/sex	Primary MALT lymphoma	Chronic disease	Dissemination	Serum Ig	CD5	Genetic findings	Reference
Levine et al./1989	56/M	Eye	—	BM	IgM*λ*	−	T(11;18)	[16]
Ueda et al./1996	48/M	Liver	—	—	IgM*κ*	+	NA	[31]
Matsumoto et al./1996	74/F	Duodenum	—	—	IgA*κ*	−	NA	[32]
Nakata et al./1997	74/M	Eyes	—	—	IgM*κ*	−	NA	[33]
Mak et al./1998	62/M	Kidney	IgA NP	GI tract	IgM*λ*	−	NA	[34]
Sakai et al./2000	72	Ileum and colon	ITP, AIH	−	IgG*κ*	−	NA	[35]
Valdez et al./2001	50/M	Nasopharynx	—	BM	IgM*κ*	−	NA	[9]
	40/M	Eye and lung	—	—	IgM*κ*	−	NA	
	60/F	Salivary gland	Gougerot syndrome	BM	IgM*κ*	−	NA	
	61/F	Lung	—	PE, skin, and pericardium	IgM*κ*	−	NA	
	74/M	Eye and pharynx	—	—	IgM, IgA*κ*	−	NA	
Nagakawa et al./2002	61/M	Lung	—	BM	IgM	NA		[36]
Pachmann et al./2002	59/	Salivary gland	—	BM, LN, kidneys, liver	IgG*λ*	−	NA	[37]
Stokes et al./2002	72/F	Kidney	MPGN	—	IgM*κ*	−	NA	[38]
Thieblemont et al./2002	60/F	Thyroid	Hashimoto	—	IgG*κ*	−	NA	[39]
Saito et al./2004	65/F	Small bowel	GN and ascariasis	Ascites	IgM*κ*	NA	T(11;18)	[40]
Takasaki et al./2005	84/M	Lung	—	BM and PE	IgM	−	T(11;18)	[41]
Dalle et al./2006	49/M	Skin	Schnitzler syndrome	BM	IgM*κ*	+	NA	[42]
Gomyo et al./2007	67/F	Pleura	—	—	IgM	−	T(14:18)	[43]
Schulze et al./2007	75/M	Lung	—	BM	IgM*κ*	−	T(11;18)	[44]
Ohno and Isoda/2008	77/M	Lung	—	BM and PB	IgM*κ*	−	T(11;18)	[27]
Murota et al./2009	73/F	Skin	Schnitzler syndrome	BM	IgM*κ*	−	NA	[45]
Mikolaenko and Listinsky/2009	75/F	Salivary gland	RA	BM and lung	IgM*λ*	+	NA	[46]
Peces et al./2010	77/M	Kidney	Barrett's esophagus	BM	IgM*κ*	−	NA	[47]
Mitchum et al./2010	46/M	Skin	—	BM	IgM*κ*	−	Bcl-2	[48]
Ikuta et al./2010	54/F	Colon	—	—	IgM*κ*	−	NA	[49]
Kim et al./2011	66/M	Small bowel	—	BM	IgM*λ*	−	NA	[50]
Lacoste et al./2013	74/F	Skin	Angiomatosis	BM and PB	IgM*κ*	+	NA	[51]
Wu et al./2014	70/M	Lung	—	—	IgA*κ*	−	NA	[30]
Chi et al./2014	72/F	Kidney	CKD	BM and PB	IgM*κ*	−	NA	[52]

NA: not available; NK: normal karyotype; PE: pleural effusion; BM: bone marrow; PB: peripheral blood; IgA NP: IgA nephropathy; ITP: idiopathic thrombocytopenic purpura; AIH:autoimmune hepatitis; MPGN: membranoproliferative glomerulonephritis; GN: glomerulonephritis; RA: rheumatoid arthritis; CKD: chronic kidney disease.
